# A Study of Solvent Effects in the Solvolysis of Propargyl Chloroformate

**DOI:** 10.5402/2011/767141

**Published:** 2011-04-11

**Authors:** Malcolm J. D'Souza, Anthony M. Darrington, Dennis N. Kevill

**Affiliations:** ^1^Department of Chemistry, Wesley College, 120 North State Street, Dover, DE 19901-3875, USA; ^2^Department of Chemistry and Biochemistry, Northern Illinois University, DeKalb, IL 60115-2862, USA

## Abstract

The specific rates of solvolysis of propargyl chloroformate (**1**) are analyzed in 22 solvents of widely varying nucleophilicity and ionizing power values at 25.0°C using the extended Grunwald-Winstein equation. Sensitivities to solvent nucleophilicity (*l*) of 1.37 and to solvent ionizing power (*m*) of 0.47 suggest a bimolecular process with the formation of a tetrahedral intermediate. A plot of the rates of solvolysis of **1** against those previously reported for phenyl chloroformate (**2**) results in a correlation coefficient (*R*) of 0.996, a slope of 0.86, and an *F*-test value of 2161. The unequivocal correlation between these two substrates attests that the stepwise association-dissociation (A_N_ + D_N_) mechanism previously proposed for **2** is also operative in **1**.

## 1. Introduction

Propargyl chloroformate (**1**) has been shown to be a very useful reagent that is used to introduce the propargyloxycarbonyl protecting group in reaction selective chemistry [1–3]. It has also found use in polymerizable acrylic compositions for the paint industry [4], and like other chloroformate esters, it could pose an environmental hazard [5] as chloroformate esters that readily react with moisture and have a corrosive effect on the human respiratory system [6].

**Figure 1:**
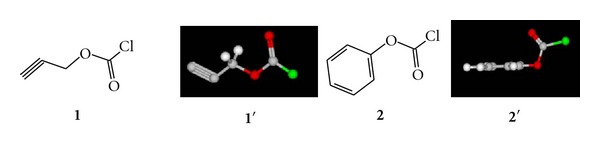
Molecular structures of propargyl chloroformate (**1**) and phenyl chloroformate (**2**) and the 3D images for the *syn* conformer of propargyl chloroformate (**1′**) and phenyl chloroformate (**2′**).

In Figure 1, the molecular structures and 3D structures of propargyl (**1,** **1′**) and phenyl (**2,** **2′**) chloroformate are shown in their most stable configuration [7, 8] where the C=O is *syn* with respect to the alkynyl or aryl moiety, that is, the halogen atom is in a *trans* position with respect to the alkynyl or aryl group. 

In physical organic chemistry, linear free energy relationships (LFERs) such as the simple (1) [9] and extended (2) [10] Grunwald-Winstein equations are utilized to evaluate solvolytic mechanisms of a variety of substrates. In (1) and (2), *k* and *k*_*o*_ are the specific rates of solvolysis of a substrate in a given solvent and in the standard solvent (80% ethanol), respectively, *m* represents the sensitivity to changes in the solvent ionizing power *Y*_X_ (based on the solvolysis of 1- or 2-adamantyl derivatives) [11–15], *l* is the sensitivity to changes in solvent nucleophilicity *N*_T_ (based on the solvolysis of *S*-methyldibenzothiophenium ion) [16, 17], and *c* is a constant (residual) term

(1) $$\begin{array}{c} \log\left(\frac{k}{k_{o}}\right)=mY_{\text{X}}+c, \end{array}$$

(2) $$\begin{array}{c} \log\left(\frac{k}{k_{o}}\right)=lN_{\text{T}}+mY_{\text{X}}+c. \end{array}$$

Equations (1) and (2) have been successfully used to correlate unimolecular ionization (S_N_1 + E1) and bimolecular nucleophilically solvent-assisted (S_N_2 and/or E2) reactions [18–22]. For compounds where resonance delocalization was possible between the reaction site and an adjacent *π*-system or for solvolyses of *α*-haloalkyl aryl compounds that proceed via anchimeric assistance (*k*_∆_), we proposed [22, 23] adding an additional term, the aromatic ring parameter *I*, to (1) and (2), to give (3). In (3), *h* represents the sensitivity of solvolyses to changes in the aromatic ring parameter *I*

(3) $$\begin{array}{c} \log\left(\frac{k}{k_{o}}\right)=mY_{\text{X}}+hI+c,\\ \log\left(\frac{k}{k_{o}}\right)=lN_{\text{T}}+mY_{\text{X}}+hI+c. \end{array}$$

**Scheme 1:**
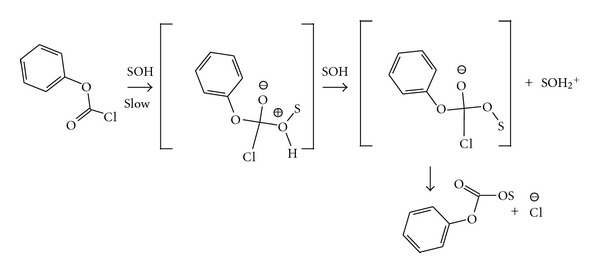
Stepwise addition-elimination mechanism through a tetrahedral intermediate for phenyl chloroformate (**3**).

In Scheme 1, we depict the solvolysis of phenyl chloroformate (PhOCOCl, **2**) with the observed sensitivity values [24, 25] of *l* = 1.66 and *m* = 0.56 utilizing the extended Grunwald-Winstein equation (2). These values were obtained over the full range of the types of solvent usually incorporated into such studies, and these *l* and *m* values are now taken as typical values [19–21, 24, 25] for attack at an acyl (sp^2^) carbon proceeding by the addition-elimination mechanism, with the addition step being rate-determining.

**Figure 2:**
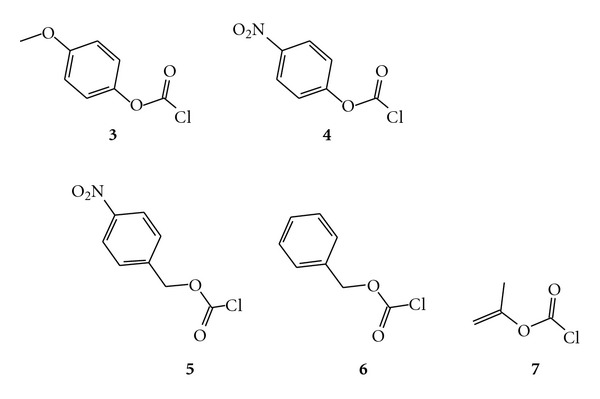
Molecular structures of *p*-methoxyphenyl chloroformate (**3**), *p*-nitrophenyl chloroformate (**4**), *p*-nitrobenzyl chloroformate (**5**), benzyl chloroformate (**6**), and isopropenyl chloroformate (**7**).

Figure 2 depicts the other aryl and alkenyl chloroformates that have been studied using (2). The aryl chloroformates *p*-methoxyphenyl (**3**) [25–28], *p*-nitrophenyl (**4**) [25, 27, 29, 30], and *p*-nitrobenzyl (**5**) [31, 32] were all shown to solvolyze like **2** by a dominant addition-elimination (A_N_ + D_N_) mechanism with rate-determining formation of a tetrahedral transition state (Scheme 1). Benzyl chloroformate (**6**) followed the A_N_ + D_N_ pathway in all binary aqueous organic mixtures except in the fluoroalcohols where a solvolysis-decomposition process was shown to be dominant [32].

The only alkenyl chloroformate studied using the extended Grunwald-Winstein analysis (2) is isopropenyl chloroformate (**7**) [33–35]. Zoon et al. analyzed the solvolyses of **7** [33] using (2) in 40 pure and binary organic mixtures at 10.0°C. Together with kinetic solvent isotope effect (KSIE) data of 2.33, they concluded [33] that the solvolytic reactions for **7** fit a third-order reaction mechanism involving attack by a solvent nucleophile assisted by another molecule of solvent acting as a general base, and the rate data could be dissected into contributions from four competing reaction channels in the alcohol-water solvent systems [33]. Koh and Kang [34] studied the solvolysis of **7** at 35.0°C in 33 solvents including the highly ionizing aqueous 1,1,1,3,3,3-hexafluoro-2-propanol (HFIP) and 2,2,2-trifluoroethanol (TFE) mixtures. On application of (2), they obtained an *l* value of 1.42 and an *m* value of 0.46 [34] and suggested that **7** solvolyzed by an addition-elimination (A_N_ + D_N_) mechanism involving rate-limiting attack by the solvent at the carbonyl carbon of **7**. With the *k*_MeOH_/*k*_MeOD_ data of 2.19 achieved [34], they inferred that a general base catalysis is also superimposed upon the A_N_ + D_N_ bimolecular process.

**Figure 3:**
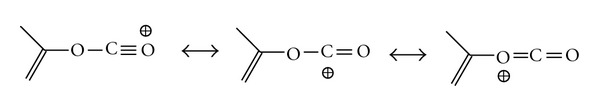
Resonance stabilized transition state of isopropenyl chloroformate (**7**).

Recently, we completed an exhaustive evaluation [35] of the solvolysis of **7** at 10.0°C in 51 solvents with widely varying nucleophilicity and ionizing power values. Outcomes acquired through the application of the extended Grunwald-Winstein equation (2) resulted [35] in the proposal of an addition-elimination (A_N_ + D_N_) mechanism dominating in most of the solvents, but in 97–70% HFIP, and 97% TFE, a superimposed S_N_1-type ionization is making a significant contribution. We proposed [35] that for the solvolysis of **7 **in 97% HFIP, 97% of the reaction undergoes solvolyses by an ionization (S_N_1) process and in 90% HFIP, 70% HFIP, and 97% TFE, the corresponding % ionization values are 70%, 64%, and 35%, respectively. We suggested [35] that such superimposed unimolecular (S_N_1) processes are observed in the highly ionizing aqueous fluoroalcohol mixtures for **7** are due to the formation of a resonance stabilized transition state shown in Figure 3.

In this paper, we will now report our analyses for the first alkynyl ester, propargyl chloroformate (**1**), to be studied using the extended Grunwald-Winstein equation (2) in a variety of mixed aqueous organic solvents at 25.0°C. Theoretically, this ester (**1**) like benzyl chloroformate (**6**) [32] could undergo heterolytic bond cleavage in a solvolysis-decomposition type process with loss of CO_2_ with the formation of a resonance stabilized intermediate.

## 2. Results and Discussion

The first-order specific rates of solvolysis for **1** were determined in 22 solvents at 25.0°C. The solvents consisted of methanol (MeOH), ethanol (EtOH), and binary mixtures of water with methanol, ethanol, acetone, TFE, or HFIP, plus binary mixtures of TFE with ethanol. These values together with the literature values for *N*_T_ [16, 17] and *Y*_Cl_ [12–15] are reported in Table 1.

A comparison of the specific rates of solvolysis for **1** (Table 1) with those previously reported for **2** [24, 25, 27] at 25.0°C gives *k*_2_/*k*_1_ ratios of 6 to 11 in the aqueous ethanol, methanol, and acetone mixtures, ratios of 2 to 4 in the more aqueous fluoroalcohols, and a ratio of 1.3 in the highly ionizing 97% HFIP. This rate sequence implies that a similar biomolecular mechanism is occurring in both substrates with the inductive effect of the phenoxy group being much greater than that of the propargoxy group. Such differences in electron withdrawing character are further corroborated by the 3D images for propargyl chloroformate (**1′**) and phenyl chloroformate (**2′**) shown in Figure 1, where due to the presence of the additional methyl group, the alkynyl group is twisted out of the plane of the ether oxygen.

**Figure 4:**
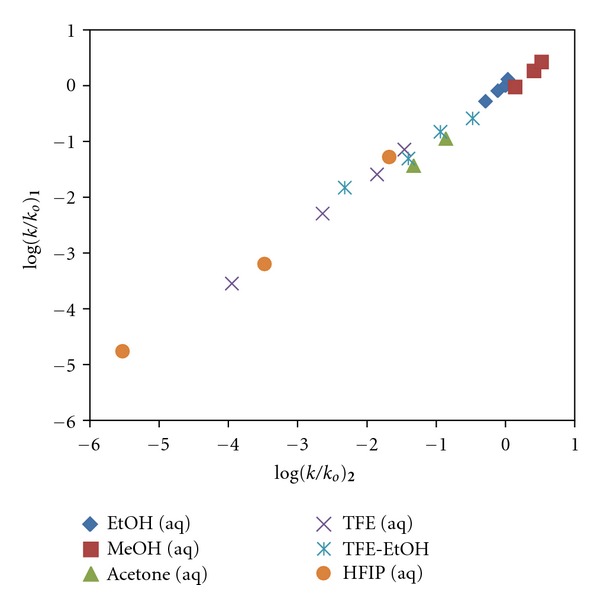
The plot of log⁡⁡(*k*/*k*_*o*_) for propargyl chloroformate (**1**) against log⁡⁡(*k*/*k*_*o*_) for phenyl chloroformate (**2**) in common pure and binary solvents at 25.0°C.

A plot of log⁡⁡(*k*/*k*_*o*_) for propargyl chloroformate (**1**) against log⁡⁡(*k*/*k*_*o*_) for phenyl chloroformate (**2**) is shown in Figure 4. This graph has an *R* value of 0.996, an *F*-test of 2161, a slope of 0.86 ± 0.02, and an intercept of −0.04 ± 0.04. These values provide strong evidence that **1** undergoes solvolysis by a similar mechanism to **2**.

In Table 2, we report the results obtained on application of the extended Grunwald-Winstein equation (2) to the specific rates of solvolysis of **1 **in all of the 22 solvents studied. We obtain an *l* value of 1.37 ± 0.10, an *m* value of 0.47 ± 0.07, an *l*/*m* ratio of 2.91, a correlation coefficient (*R*) of 0.970, an *F*-test value of 152, and an intercept of 0.11 ± 0.11. The *l*/*m* ratio of **1** in 22 solvents is similar to that reported for **2** [24, 25] in 49 solvents (Table 2).

In Table 2, we also report the analyses obtained for **1 **using (2) in 20 solvents (no 95% acetone, 80% HFIP). We report 1.44 ± 0.11 for *l*, 0.51 ± 0.08 for *m*, an *l*/*m* ratio of 2.82, *R* = 0.977, an *F*-test value of 181, and *c* = 0.12 ± 0.10. For **2** in the identical 20 solvents, we get 1.55 ± 0.13 for *l*, 0.48 ± 0.09 for *m*, an *l*/*m* ratio of 3.23, *R* = 0.978, *F*-test = 186, and an intercept of 0.14 ± 0.12. These statistical values coupled with the data reported above for Figure 4, strongly demonstrates that **1** and **2** undergo a very similar bimolecular addition-elimination (A_N_ + D_N_) process with the addition-step being rate determining. 

The solvolyses of **7** at 25.0°C were studied [35] in 100% EtOH (110 ± 6 × 10^−5^ s^−1^), 100% MeOH (210 ± 8 × 10^−5^ s^−1^), 70% HFIP (2.54 ± 0.09 × 10^−5^ s^−1^), and 50% HFIP (35.2 ± 3.1 × 10^−5^ s^−1^). The corresponding *k*_7_/*k*_1_ ratios in the common solvents studied are 3.14 in pure EtOH, 3.31 in 100% MeOH, and 0.18 in 70% HFIP. These results showing only small differences between *k*_7_ and *k*_1_ in MeOH and EtOH affirm the proposal [35] that **7** undergoes solvolysis by a stepwise addition-elimination (A_N_ + D_N_) with a rate-determining addition step. The rates of solvolysis of **7 **are 3-fold faster in MeOH and EtOH when compared to those of **1** due to the proximity of the alkenyl group and the ether oxygen in **7** and the fact that the alkynyl group is pushed out of the plane of the ether oxygen in **1**. 

**Figure 5:**
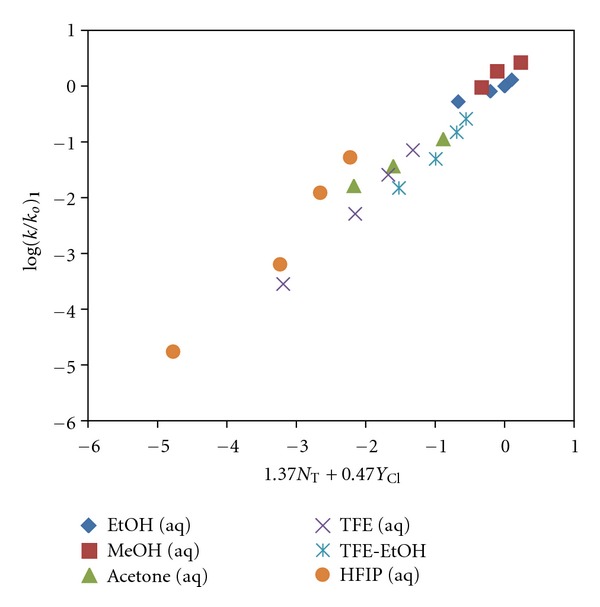
The plot of log⁡⁡(*k*/*k*_*o*_) for propargyl chloroformate (**1**) against 1.37*N*_T_ + 0.47*Y*_Cl_.

A plot of log⁡⁡(*k*/*k*_*o*_) for propargyl chloroformate (**1**) against 1.37*N*_T_ + 0.47*Y*_Cl_ shown in Figure 5 shows that the 97% HFIP and 90% HFIP points lie slightly above the regression line. Removing of these two data points and on using (2) in the remaining 20 solvents, we get an *l* value of 1.33 ± 0.13, *m* = 0.46 ± 0.07, *R* = 0.944, *F*-test = 69, and *c* = 0.09 ± 0.12. The much lower *R* and *F*-test values obtained using these 20 solvents when compared to those obtained with (2) using all of the 22 solvents studied (Table 2) suggest that the plot shown in Figure 5 is robust and that the addition-elimination (A_N_ + D_N_) process dominates in all of the 22 solvents studied. 

## 3. Conclusions

The mechanism of reaction for the solvolysis of propargyl chloroformate (**1**) in all 22 solvents with widely ranging nucleophilicity and ionizing power values is found to closely mimic that of the previously studied phenyl chloroformate (**2**). For **1** in all 22 solvents, we propose an addition-elimination (A_N_ + D_N_) process with the addition-step being rate determining.

The *k*_2_/*k*_1_ rate ratios suggest that the inductive ability of the alkynoxy group in **1 **is reduced because the alkynyl group is pushed out the plane of the ether oxygen. The extended Grunwald-Winstein equation (2) is again shown to be very sensitive in deciphering solvents effects. 

## 4. Experimental Section

The propargyl chloroformate (Sigma-Aldrich, 97%) was used as received. Solvents were purified and the kinetic runs carried out as described previously [24]. A substrate concentration of approximately 0.005 M in a variety of solvents was employed. The specific rates and associated standard deviations, as presented in Table 1, are obtained by averaging all of the values from, at least, duplicate runs.

Multiple regression analyses were carried out using the Excel 2007 package from the Microsoft Corporation. The 3D-views presented in Figure 1 were computed using the KnowItAll Informatics System, ADME/Tox Edition, from BioRad Laboratories, Philadelphia, Pa, USA.

## Figures and Tables

**Table 1:** Specific rates of solvolysis (*k*) of **1**, in several binary solvents at 25.0°C and the literature values for (*N*_*T*_) and (*Y*_*Cl*_).

Solvent (%)^a^	1 at 25.0°C; 10^5^ *k*, s^−1 b^	*N*_T_^ c^	*Y*_Cl_^ d^
100% MeOH	63.4 ± 1.2	0.17	−1.2
90% MeOH	123 ± 3	−0.01	−0.20
80% MeOH	178 ± 10	−0.06	0.67
100% EtOH	35.0 ± 0.8	0.37	−2.50
90% EtOH	53.9 ± 1.2	0.16	−0.90
80% EtOH	66.7 ± 1.6	0.00	0.00
70% EtOH	86.7 ± 1.7	−0.20	0.80
95% Acetone	1.09 ± 0.03	−0.49	−3.19
90% Acetone	2.46 ± 0.10	−0.35	−2.39
80% Acetone	7.52 ± 0.22	−0.37	−0.80
97% TFE (w/w)	0.0190 ± 0.0007	−3.30	2.83
90% TFE (w/w)	0.342 ± 0.007	−2.55	2.85
80% TFE (w/w)	1.72 ± 0.01	−2.22	2.90
70% TFE (w/w)	4.78 ± 0.07	−1.98	2.96
80T-20E	0.995 ± 0.004	−1.76	1.89
60T-40E	3.31 ± 0.01	−0.94	0.63
40T-60E	10.0 ± 0.2	−0.34	−0.48
20T-80E	17.4 ± 1.0	0.08	−1.42
97% HFIP (w/w)	0.00116 ± 0.00009	−5.26	5.17
90% HFIP (w/w)	0.0426 ± 0.0020	−3.84	4.41
80% HFIP (w/w)	0.821 ± 0.003	−3.31	3.99
70% HFIP (w/w)	13.9 ± 0.8	−2.94	3.83

^a^Substrate concentration of *ca.* 0.0052 M, binary solvents on a volume-volume basis at 25.0°C, except for TFE-H_2_O and HFIP-H_2_O solvents which are on a weight-weight basis. T-E are TFE-ethanol mixtures. ^b^With associated standard deviation. ^c^References [16, 17]. ^d^References [12–15].

**Table 2:** Correlation of the specific rates of reaction of **1**–**7** using the extended Grunwald-Winstein equation (2).

Substrate	*n* ^ a^	*l* ^ b^	*m* ^ b^	*l*/*m*	*c* ^ c^	*R* ^ d^	*F* ^ e^
**1**	22	1.37 ± 0.10	0.47 ± 0.07	2.91	0.11 ± 0.11	0.970	152
	20^f^	1.44 ± 0.11	0.51 ± 0.08	2.82	0.12 ± 0.10	0.977	181
**2**	49^g^	1.66 ± 0.05	0.56 ± 0.03	2.96	0.15 ± 0.07	0.980	568
	20^h^	1.55 ± 0.13	0.48 ± 0.09	3.23	0.14 ± 0.12	0.978	186
**3**	44^i^	1.60 ± 0.05	0.57 ± 0.05	2.81	0.18 ± 0.06	0.981	517
**4**	39^j^	1.68 ± 0.06	0.46 ± 0.04	3.65	0.074 ± 0.08	0.976	363
**5**	19^k^	1.61 ± 0.09	0.46 ± 0.04	3.50	0.04 ± 0.22	0.975	157
**6**	15^l^	1.95 ± 0.16	0.57 ± 0.05	3.42	0.16 ± 0.15	0.966	83
	11^l^	0.25 ± 0.05	0.66 ± 0.06	0.38	−2.05 ± 0.11	0.976	80
**7**	50^m^	1.54 ± 0.06	0.54 ± 0.03	2.85	0.05 ± 0.06	0.968	347

^a^ *n* is the number of solvents. ^b^With associated standard error. ^c^Accompanied by standard error of the estimate. ^d^Correlation coefficient. ^e^ *F*-test value. ^f^No 95A, 80HFIP, to compare with **2** in identical solvents. ^g^Values taken from [24, 25]. ^h^To compare with **1** in identical solvents. ^i^Values taken from [25, 28]. ^j^Values taken from [30]. ^k^Values taken from [31, 32]. ^l^Values taken from [32]. ^m^Values taken from [35].
